# The cardiovascular system and the biochemistry of grafts used in heart surgery

**DOI:** 10.1186/2193-1801-2-612

**Published:** 2013-11-16

**Authors:** Suna Aydin, Suleyman Aydin, Mehmet Nesimi Eren, İbrahim Sahin, Musa Yilmaz, Mehmet Kalayci, Orhan Gungor

**Affiliations:** Elazig Research and Education Hospital, Clinic of Cardiovascular Surgery, 23119 Elazig, Turkey; School of Medicine, Department of Anatomy, Firat University, 23119 Elazig, Turkey; School of Medicine, Department of Medical Biochemistry (Firat Hormone Research Groups), Firat University, 23119 Elazig, Turkey; School of Medicine, Department of Cardiovascular Surgery, Dicle University, 21280 Diyarbakir, Turkey; School of Medicine, Department of Histology & Embryology, Erzincan University, 24030 Erzincan, Turkey

**Keywords:** Cardiovascular system, Graft, Blood vessels, Coronary artery bypass

## Abstract

Blood is pumped into the cardiac muscle through arteries called the coronary arteries. Over time, the accumulation of cholesterol, coagulation factors, and cells on the walls of these arteries causes the walls to thicken and lose their elasticity, resulting in the development of atherosclerosis. When the blood supply of the heart is diminished by atherosclerosis, it can be restored by bypass surgery, in which atherosclerosis-free vein and/or artery grafts taken from another area of the body are used to replace the atherosclerotic vessels. These biological grafts used in surgery differ in biochemical composition and long-term patency. Although the great saphenous vein (GSV) has been the most popular graft material in revascularization for years, it has recently been superseded by the internal mammarian artery (IMA), which has a lower incidence of recurrence of atherosclerosis. The aim of the present review is briefly to address the structure of the cardiovascular system and blood vessels, and then, in the light recent data, to present the biochemical compositions and individual advantages of the graft materials used to restore an impaired blood supply to the heart.

**Electronic supplementary material:**

The online version of this article (doi:10.1186/2193-1801-2-612) contains supplementary material, which is available to authorized users.

## Introduction

### The cardiovascular system and the heart

The major component of the cardiovascular system is the heart, a muscular structure that pumps blood into two distinct circulatory systems, namely the pulmonary circulation that carries blood to and from the lungs, and the systemic circulation that transports blood to and from all the tissues and organs of the body (Norman and Litwack 1997;Skwarek et al. 2006;Aydin 2011). An adult heart is about the size of a person’s clenched fist and weighs about 280 to 340 g in men and 230 to 280 g in women (Norman and Litwack 1997). The human heart, which beats about 100,000 times a day, pumps 7000 to 8000 liters of blood to a blood vessel network of 96,560 kilometers in the process (Norman and Litwack 1997; About Arrhythmia [homepage on the Internet] (2012)).

The human heart, divided into right and left parts longitudinally, is also separated into ventricles and atria. The heart functions to pump the blood from the venous system to the arterial system (Norman and Litwack 1997;Skwarek et al. 2006). The right part of the heart receives deoxygenated blood from the peripheral tissues through the venae cavae and pumps it into the pulmonary artery and thence the pulmonary circulation. The left part of the heart receives oxygenated blood via the pulmonary veins and then distributes it to all the peripheral tissues through the aorta (Skwarek et al. 2006;Elghobary and Légaré^2010^). While the heart is thus employed, the coronary arteries can become constricted due to atherosclerosis and their blood supply is impaired (Elghobary and Légaré 2010;Miller et al. 2010).

Although the fact that constriction of coronary arteries due to atherosclerosis affects human health has been known for at least 3000 years, as evidenced by ancient Egyptian papyri, it has been less than 300 years since angina pectoris was first associated with coronary artery constriction and less than 100 years since the first clinical myocardial infarction was diagnosed (Cullen et al. 2005). Atherosclerosis is characterized by the accumulation of cholesterol, coagulation factors and cells on the vessel wall, weakening the underlying tunica media layer, followed by the formation of intimal atheromatous plaques, which bring about a series of complications (Elghobary and Légaré 2010;Miller et al. 2010;Cullen et al. 2005). These plaques cause the vessel walls to thicken, whereby the lumen is narrowed, reducing the blood flow. Although atherosclerosis can affect all arteries, the most commonly injured vessels are the aorta and the coronary and cerebral arteries (Cullen et al. 2005).

A heart with constricted vessels cannot function properly for long: its contraction starts to deteriorate, and this condition needs to be remedied by coronary artery bypass surgery. In this treatment, artery or vein graft materials are used to restore the blood supply to the myocardium, the area affected by atherosclerosis of the coronary artery (Aydin 2011;Elghobary and Légaré^2010^). The grafting in this operation is performed by stopping the heart, a “cardiopulmonary bypass”; or, if the grafting is carried out without stopping the heart, or off pump, the procedure is called “beating heart technique” (Skwarek et al. 2006;Creswell et al. 2005).

The aim of this review is briefly to address the structures of the blood vessels in general, and then to present a comparison of the individual advantages of internal mammarian artery (IMA) and great saphenous vein (GSV) grafts, the materials most commonly used for bypass surgery, from biochemical and physiological aspects in the light of recent data.

### Blood vessels

The other components of the cardiovascular system are the blood vessels (Skwarek et al. 2006;Aydin 2011;Cullen et al. 2005). Although blood vessels differ in some ways they display important similarities. The walls of high-pressure vessels (e.g. subclavian arteries) are thicker than those of the vessels that transport blood under lower pressure (e.g. subclavian veins). While the diameters of arteries become smaller at each branching, the diameters of veins increase with each new addition. Even though the wall structure of minor vessels such as capillaries and venules is simpler, all vessel walls are composed of three layers (Tennant and McGeachie 1990). Blood vessels are classified into six groups: arteries, arterioles, veins, venules, capillaries, and lymphatic vessels (Tennant and McGeachie 1990;Gartner and Hiatt 1997;Lafleur et al. 2003). The structures of these vessels will be addressed in general; individual groups of vessels will not be described separately. However, the vessels used for grafting in coronary artery bypass surgery will be dealt with in detail where appropriate in later parts of this review.

### The structure of blood vessels

**Figure 1:**
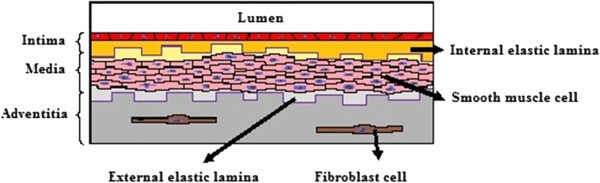
**The histological structure of blood vessels.** Bloocd vessels are composed of three layers, called (from the lumen outwards) intima, media, and adventitia.

Blood vessel walls comprise three main strata, namely the tunica intima, tunica media, and tunica adventitia (Figure 1). These three strata are described below (Tennant and McGeachie 1990).

#### Tunica intima (inner layer)

The innermost portion of the wall is a single-cell endothelial layer attached to a basal lamina. Among other products, endothelial cells secrete type II, IV and V collagens, laminin, endothelin, nitric oxide, and von Willebrand factor. The subendothelial layer comprises the basal lamina and a loose, fibroelastic band of connective tissue and occasional smooth muscle cells. Both these layers are arranged longitudinally. The membrana elastica interna is the outermost portion of the subendothelial layer, where elastic fibers are concentrated (Tennant and McGeachie 1990;Gartner and Hiatt 1997;Lafleur et al. 2003).

#### Tunica media (middle layer)

This layer is composed of smooth muscle cells in a circular arrangement, arranged concentrically on a matrix containing type III collagen with proteoglycans together with elastic fibers and elastic membranes. This layer, which is very well developed in arteries, is formed from extracellular matrix smooth muscle cells. Capillaries and postcapillary venules have no tunica media layer (Tennant and McGeachie 1990;Gartner and Hiatt 1997).

#### Tunica adventitia (outer layer)

The outermost layer is composed of collagen and elastic fibrils. In the veins, it consists mainly of smooth muscle. The elastic fibrils in this area are concentrated to form the membrana elastica externa. The adventitia is the most prominent layer in vein walls (Tennant and McGeachie 1990;Gartner and Hiatt 1997;Lafleur et al. 2003). It extends into the peripheral connective tissue. The walls of large vessels are perfused by small blood vessels, the vasa vasorum, within the adventitia; the adventitia and media layers are too thick to be supplied with nutrients and oxygen by diffusion from the lumen. These vessels are found in smaller numbers in the arteries but are more numerous and can extend to the media layer in the veins. The intima and the innermost part of the media in veins contain no vasa vasorum. Similarly, lymphatic capillaries are found only in the adventitia in arteries, but can reach the media layer in veins (Tennant and McGeachie 1990).

Smooth muscles in blood vessel walls are stimulated by unmyelinated sympathetic nerves (vasomotor nerves), which contain the vasoconstrictor norepinephrin. Cholinergic vasodilator nerves also supply the arteries in skeletal muscle tissue. To effect contraction of the arterial wall smooth muscle, norepinephrin must diffuse into the media layer. Gap junctions between the smooth muscle cells in the tunica media layer enable neurotransmitters to be transported to the inner muscle cells. While there are nerve endings in both the adventitia and media layers in the veins, arteries in general are more richly supplied with nerve cells (Gartner and Hiatt 1997).

### Coronary artery bypass surgery graft materials

A coronary bypass operation is performed on about one million individuals around the world per annum. Gordon Murray, who studied the suturing of mammarian, axillary or carotid arteries on the diseased left anterior descending (LAD) artery in 1953, was the first to show the efficacy of arterial grafts in the coronary circulation (Murray 1953). Four years later, Sidney Smith reported that a segment of saphenous vein from the leg could be used to create a direct bypass from the aorta to the myocardium and could maintain coronary blood circulation (Smith et al. 1957). Currently, arterial grafts, venous grafts, and synthetic grafts are all used in coronary bypass surgery. Grafts are usually classified as autologous and non-autologous. Non-autologous arterial (pig internal thoracic artery (ITA)) and non-autologous venous (v. umbicalis, VSM homograft) grafts are used, but not commonly, so they will not be addressed in this article. Autologous arterial grafts used in coronary bypass surgery include those from the arteria radialis (AR), arteria gastroepiploica dextra (AGED), arteria epigastrica inferior (AEI), a. splenica, a. ulnaris, a. subscapularis, gastric sinistra, and a. circumflexa femoris lateralis (Tennant and McGeachie 1990;Deppe et al. 2013). However, since long-term patency is critical for aorta coronary bypass operations, the LIMA and saphena, which have longer patency, are the two most commonly used graft materials at present (Ferrari and von Segesser 2006). The patency rate for AR, for instance, is reported to be 98% initially, but decreases to 90% after one year (Bleiziffer et al. 2007;Erenturk 1997). When the post-operative ten-year patency rates for the left ITA AR and VSM were compared angiographically, they were reported to be 97.5% for the left ITA 91.6% for the AR, and 67.1% for the VSM (Risteski et al. 2006). Therefore, the present study will focus primarily on the left internal mammarian artery (LIMA) and the greater saphenous vein (GSV).

### Arteries

The diameters of the arteries that transport the blood to the capillary beds decrease with increasing distance from the heart. Two arteries branch out from the heart. One is the truncus pulmonalis, which arises from the right ventricle; the other is the aorta, arising from the left ventricle. The aorta, which arises from the upper part of the heart, then makes a turn to the left and descends (Tennant and McGeachie 1990;Lafleur et al. 2003). The internal mammarian artery is the major graft material in heart surgery (Erenturk 1997).

#### Left internal mammarian artery (LIMA)

The left internal mammarian artery (LIMA) arises from the early inferior part of the subclavian artery in the medial segment of the anterior scalene muscle. Passing between the clavicula and the first costal cartilage, it descends to the thorax and proceeds along the lateral side of the sternum (Tennant and McGeachie 1990;Gartner and Hiatt 1997). Posteriorly, it is above the pleura and is traversed by the phrenic nerve. It proceeds behind the superior sixth costal cartilage between the intercostal muscles. After it passes the third costal cartilage it moves to the anterior side of the transversus thoracis muscle and then bifurcates into the superior epigastric and musculophrenic arteries at the sixth costal cartilage (Tennant and McGeachie 1990;Gartner and Hiatt 1997;Lafleur et al. 2003). A bypass of the LIMA to the LAD artery is common (Erenturk 1997;Sabik et al. 2008). Use of the left internal thoracic artery instead of the saphenous vein in coronary artery bypass surgery was widely accepted in angiographic studies during the 1980s (Fukui et al. 2010). Similarly, the use of bilateral LIMA as a graft material was reported to improve survival in recurrent myocardial ischemia after coronary bypass (Lytle et al. 1999). When the LIMA is used in coronary artery bypass grafting operations, operative mortality is reduced, except for mortality due to complications associated with the operation (Dabel et al. 2003). A LIMA bypass to the left descending coronary artery in particular has long-term patency rates. Also, when the LIMA was used instead of the saphenous vein for grafting to the LAD artery, positive effects on long-term morbidity and mortality were reported (Crouch et al. 1999). When the GSV was used as the graft material, it was reported to provide a higher flow capacity than the in situ LIMA (Fichelle et al. 2010). Histologically, the LIMA contains fewer smooth muscle cells and has a more elastic structure (Tennant and McGeachie 1990). Anastomosis of the left ITA to the RIA in coronary bypass operations is the preferred anastomosis (Fukui et al. 2010); it is accepted world-wide as the “gold standard”.

#### Radial artery

Conventional CABG surgery utilizes a combination of arterial and venous grafts. However, the radial artery (RA) as a coronary artery bypass graft has also grown over recent years. Because endothelial function is preserved in RA grafts (Verma et al. 2004). The RA was initially used for coronary revascularization by Carpentier and colleagues in 1971 (Carpentier et al. 1973). But, 2 years later, its use was abandoned because of an occlusion rate that was greater than that observed in saphenous vein grafts. In 1992, Acar and colleagues indicated that the RA is a reasonable alternative to other types of conduits to complement the LIMA (Acar et al. 1992).

RA use is safe in patients with moderate to severe left ventricular dysfunction (Fazel et al. 2003) and in patients over the age of 65 (Modine et al. 2002). Since RA grafts have a capable of autoregulating its size to adapt to the target coronary circulation, as was compared with GSV grafts and left internal mammary artery graft (Chong et al. 2006). Finally, heart surgeons should keep in mind that the RA should be limited to grafting native vessels with a high degree of stenosis (>70%) because of graft sensitivity to competitive flow and its increased propensity to spasm (Maniar et al. 2002).

### Veins

The saphenous vein, used as the graft material in coronary artery bypass operations, belongs to the group of superficial veins (Tennant and McGeachie 1990). There are two main superficial veins in the lower extremities, the vena saphena magna and vena saphena parva. Since they are located above the membranous superficial fascia and not in the skeletal muscle, these superficial veins lack supporting tissue (Gartner and Hiatt 1997;Lafleur et al. 2003). The vena saphena magna originates from the dorsal venous arch as the continuation of the v. marginalis medialis in the dorsal part of the foot. It passes the anterior side of the malleoli, proceeds inside the calf, and, after passing through the posteromedial segment of the popliteal cavity, reaches the interior aspect of the femur (Tennant and McGeachie 1990). Then it goes upwards and flows into the femoral vein in the fossa ovalis at the superficial inguinal level. The vena saphena magna is connected to the deep venous system and the vena saphena parva through the v. perforans. The branch that is critically involved in the formation of varicose masses, and flows into the vena saphena magna, is the posterior arch vein (also known as the Leonardo vein), which drains three Cockett’s perforator vein groups (Taggart et al. 2001).

### Vena saphena magna graft

The distal or proximal saphenous vein is commonly used for coronary artery bypass operations. The saphenous vein is preferred in grafting because its femoral segment is larger and it has a thick surface. J. Kunlin was the first to use the saphenous vein as the graft material in femoro-popliteal bypass in different locations in 1950 (Kunlin et al. 1950). The quality, length, histological structure, and the status of its endothelial cells all contribute to the usefulness of the saphenous vein as a graft material (Crouch et al. 1999)]. The success of the saphenous vein as the graft material in aorto-coronary bypass is reported to depend on the presence of perivenous fat and lack of traumatic injury (Erenturk 1997). It has also been noted that the administration of papaverine to the peripheral tissues around the saphenous vein increased its patency (Kocailik et al. 2008). Venous grafts are used in a variety of locations. A femoro-popliteal bypass can be used in the reverse direction, or in the same direction after the valves are removed (Erenturk 1997;Alrawi et al. 2001). Furthermore, the great saphenous vein or the small saphenous vein, as well as veins taken from the arm, can be used as graft materials for vessels around the tibia, ankle, and foot. The saphenous vein has become indispensable as graft material for hepatic-renal, iliorenal and aorto-coronary bypasses, when it is available for use (Kelsall et al. 2005). It is also the graft material of choice in cases of carotid narrowing where endarterectomy is not possible (Norman and Litwack 1997). No-touch techniques have been developed to remove the saphenous vein with as little injury as possible. Uninjured saphenous veins with a diameter of 5 mm taken from the tibial area and including as few valves as possible are preferred as high-quality graft materials (Kelsall et al. 2005;Plass et al. 2010). Studies evaluating the effects of neuropeptides including substance P, vasoactive intestinal peptide, calcitonin gene-related peptide, neuropeptide Y and somatostatin on the human saphenous vein showed many effects on vascular tonus and these effects were endothelium-dependent (Luu et al. 1992). Further studies are suggested to explore how these effects can increase the performance of graft materials in bypass operations.

### Comparison of grafts in terms of biochemical composition

Until two decades ago, the autogenous greater saphenous vein (GSV) was the most popular graft material in bypass surgery for atherosclerotic coronary artery disease, despite the common occurrence of atherosclerotic events in the post-operative period. However, recent studies have reported that, owing to its long-term patency or the late development of atherosclerotic lesions, the internal mammary artery (IMA) could be the best alternative for revascularization (Erenturk 1997;Sisto et al. 1990). The later development of atherosclerotic lesions in the IMA than in the saphenous vein has been attributed to the difference in biochemical compositions of the two vessels (Table 1).

**Table 1:** **Comparison of some biochemical compositions of internal mammary artery and greater saphenous vein grafts (Skwarek et al.** 2006 **; Sisto et al.** 1990 **; Hwang et al.** 2012 **; Oku et al.** 1990 **; Vanhoutte** 1997 **); Aydin** 2011 **; Aydin et. al** 2012

Molecules	Internal mammary artery (IMA)	Greater saphenous vein (GSV)
Heparan sulfate	High	Low
Dermatan sulfate	Low	High
Esterified cholesterol	High	Low
Free cholesterol	High	Low
Collagen	Low	High
Phospholipids	High	Low
DNA	High	Low
Protein	High*	Low
Total GAG	High	Low
Hyaluronic acid	High	Low
Chondroitin sulfate (A + B)*	High	Low
Nitric oxide (NO)	High	Low
Salusin-α	Low	High
Salusin-β	High*	Low
Apelin-36	High*	Low

*High, but not significant.

The endothelial cells of arteries have a greater capacity to secrete endothelium-dependent relaxation factors (EDRF) than those of veins. The main functions of EDRF include endothelium-dependent relaxation, prevention of vasospasms, and protection against intravascular thrombus formation and atherogenesis. The endothelium has the capacity to synthesize nitric oxide (NO), prostacyclin (PGI_2_), endogenous vasoactive or inactive products (angiotensin-1 and bradykinin), endothelin-1, and endoperoxides (prostacyclin H_2_ and thromboxane A_2_, which are constriction factors) (Kocailik et al. 2008). The endothelium of the IMA spontaneously secretes more NO than that of the GSV (Oku et al. 1990). NO, a vasodilator inhibits thrombocyte adhesion and aggregation and is critical for maintaining the patency of the vessel lumen (Kocailik et al. 2008). Furthermore, the IMA contains more elastic lamina in its tunica media than other arterial grafts or the GSV. The greater sensitivity of the IMA than the GSV to vasoactive materials is believed to be the reason for its better patency rate (Erenturk 1997;Sisto et al. 1990).

Veins are more vulnerable than arteries to vasoactive substances and cannot work under high pressure. Additionally, while vein walls are supplied by vasa vasorum, arterial walls are supplied by both the lumen and vasa vasorum. Veins secrete vasoactive substances in very different kinds and amounts from arteries (Tennant and McGeachie 1990;Gartner and Hiatt 1997;Lafleur et al. 2003). The peptides apelin and salusin, for instance, are reported to be produced in larger quantities in the aorta and LIMA than in the saphena. Salusins suppress ventricular performance and exert inotropic and chronotropic effects (Skwarek et al. 2006;Vanhoutte 1997;Aydin et al. 2012). It has been claimed that salusin β influences cardiac output more strongly than other well-known factors such as atrial natriuretic peptide or NO (Kocailik et al. 2008). These substances, which are produced in larger amounts in the arteries, could help to explain why artery grafts last longer than saphenous grafts. The major finding in support of this hypothesis is that synthetic grafts, which lack these peptide hormones, are less durable than biological grafts.

Biological grafts synthesize many substances including cholesterol, hyaluronic acid, heparan sulfate, dermatan sulfate, collagen, apelins and salusins (vasodilators). Collagen is the main structural connective tissue protein in blood vessels. The development of atherosclerosis leads to an increase in the amount of collagens in the artery walls. In the IMA, collagen constitutes between 25 and 40% of the total protein (Sisto et al. 1990). The fact that coronary arteries contain more collagen than the IMA suggests a predisposition to atherosclerosis. Of the total protein in the saphenous veins, 65% is collagen, which is much more than in arteries, another possible reason why the IMA is advantageous over the saphena (Sisto et al. 1990). Furthermore, the IMA contains less free cholesterol than the saphena. Since less cholesterol implies postponement of atherosclerosis, this could be another advantage of using the IMA. Likewise, the IMA contains more heparan sulfate than the saphena (Erenturk 1997). As heparan sulfate is associated with the bases of the cell membranes, arteries possess more cellularity than veins. In contrast, there is more dermatan sulfate in GAGs in the saphena than the LIMA. Since dermatan sulfate has a greater affinity for LDL and VLDL than other GAG components, veins are more predisposed to atherosclerosis than arteries (Sisto et al. 1990). Since the IMA secretes more prostacyclin than the SVG, platelet adhesion and activation, and vasospasms, are prevented in the former, and the root cause of atherosclerosis and graft occlusion is eliminated (Erenturk 1997). Another reason why the IMA graft is advantageous is that the diameter of this graft is compatible with the diameters of the coronary arteries. Thus, not only is turbulence eliminated, but also the incidence of thrombus development is reduced (Skwarek et al. 2006;Vanhoutte 1997). In consideration of the differences in composition between these arteries and veins, when biological grafts are going to be used for coronary bypass surgery, care should be taken to avoid injury to the endothelial layers of the vessels, where bioactive materials are synthesized. Any disruption of this layer will reduce the success of the graft. It is believed that if the cells synthesizing bioactive hormones are totally destroyed, the success rate of grafting will theoretically fall to that of synthetic grafting (Skwarek et al. 2006).

From a histopathological perspective, intimal hyperplasia, the first phase of atherosclerosis, is caused by smooth muscle cells that pass through the defects in the lamina elastica interna and invade the tunica intima. Therefore, the lamina elastica interna plays a key role in the artery wall structure, and its continuity along the vessel wall forms a barrier preventing the invasion of smooth muscle cells (Tennant and McGeachie 1990;Gartner and Hiatt 1997;Lafleur et al. 2003). This feature also makes the IMA advantageous over the GSV. However, pressure applied during harvesting of the saphenous vein graft in coronary artery bypass surgery decrease the patency (Ozturk et al. 2013).

In summary, currently most centers use the left internal thoracic (mammary) artery (LITA or LIMA). In contrast to SV grafts, the IMA displays growth potential in children with Kawasaki disease (Suda et al. 2000). IMA also has atherosclerosis preventive effects on native coronary artery which was grafted (Otsuka et al. 2013). The anastomosed site stenosis with IMA had self-healing potential and showed improved clinical outcomes. Early adaptation of ITA after CABG within a few weeks was also obtained. Arterial grafts are being increasingly used in place of the SV to avoid the late complications of vein graft atherosclerosis and restenosis. (Otsuka et al. 2013).

## Conclusion

Despite advances in technology and much hard work in the field, it has not been possible to develop graft materials that can replace natural materials. Although vein grafts are used commonly in coronary artery *bypass surgery*, the ideal graft material is the autologous LIMA. The ten-year patency rates range between 80 and 90% for the IMA and 40 and 55% for the SVG (Erenturk 1997). The difference can be explained by the different amounts of biochemical molecules in the grafts such as cholesterol, collagen, and heparan sulfate, as well as the histological compatibility and diameters of the vessels (Sisto et al. 1990;Hwang et al. 2012;Oku et al. 1990;Vanhoutte 1997). This review presents the most recent histological and biochemical data showing that IMA is the best choice in coronary bypass surgery, and could serve as a guide for heart surgeons.

## Authors’ original submitted files for images

Below are the links to the authors’ original submitted files for images. Authors’ original file for figure 1
