# Supplementary figures and images for: The cardiovascular system and the biochemistry of grafts used in heart surgery
Source: Springerplus. 2013 Nov 16;2(1):612. doi: 10.1186/2193-1801-2-612 (PMC3855918; doi:10.1186/2193-1801-2-612)

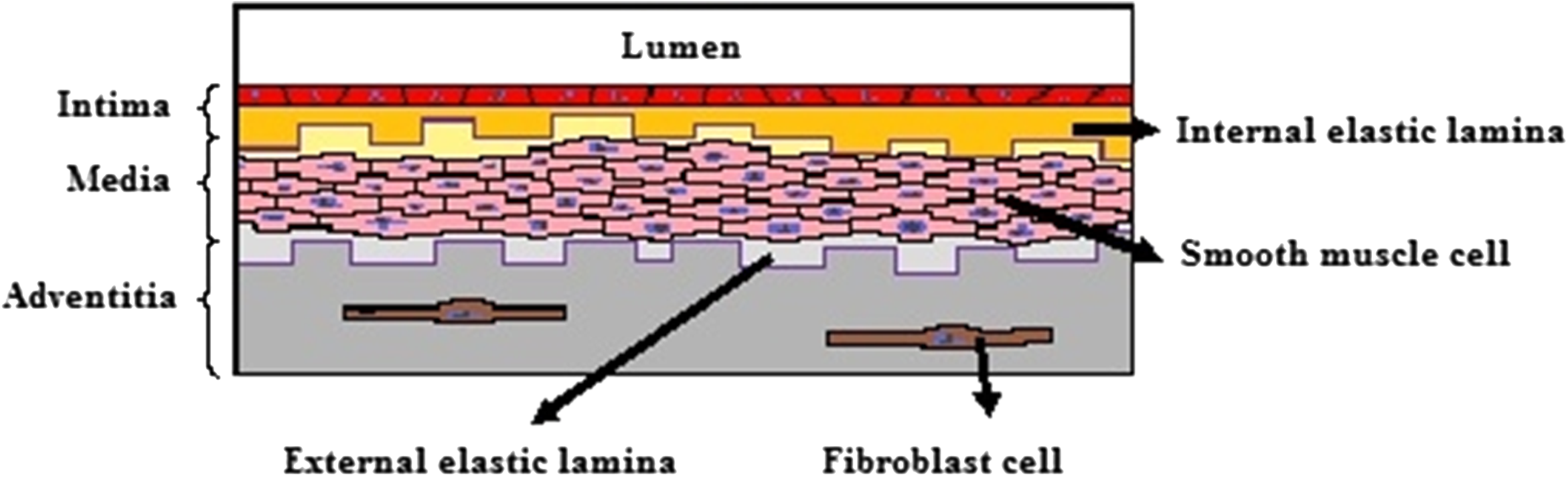

Supplement: Supplementary file 1 — Authors’ original file for figure 1 [file 40064_2013_678_MOESM1_ESM.tiff]
